# Recombinant ADAMTS 13 in thrombotic thrombocytopenic purpura

**DOI:** 10.18632/oncoscience.380

**Published:** 2017-11-28

**Authors:** Marie Scully, Christopher Hibbard, Bruce Ewenstein

**Affiliations:** Department of Haemotology; University College London Hospitals NHS Trust, Cardiometabolic Programme-NHR UCLH/UCL BRC, London, UK

**Keywords:** congenital TTP, recombinant ADAMTS 13, phase 1 study

Acute Thrombotic Thrombocytopenic Purpura (TTP) is a rare haematology emergency, associated with multi organ microthrombi formation and a high mortality if untreated. Most cases are immune mediated. Congenital TTP, thought to affect 1/million of the population, is associated with inherited homozygous or compound heterozygous defects resulting in an absolute enzyme deficiency. Congenital TTP may present in the neonatal period/childhood or delayed until adulthood, primarily pregnancy.

Currently, therapy requires replacement of the missing enzyme, ADAMTS 13 (a disintegrin and metalloproteinase with a thrombospondin type 1 motif, member 13). ADAMTS 13 is a zinc containing metalloproteinase enzyme with a unique relationship in ensuring haemostasis with its substrate VWF. Cleavage of UL VWF (ultra large von Willebrand factor) multimers by ADAMTS 13, results in haemostatically active forms. A deficiency of ADAMTS 13, as in congenital TTP or inhibition due to immune mediated disease, is associated with severely low ADAMTS 13 activity levels (<10%). This results in excess platelet binding to UL VWF multimers and resultant microvascular thrombi.

An enzyme deficiency in TTP was only confirmed 20 years ago [1, 2] and characterisation of ADAMTS 13 in 2001[3]. But therapy has little changed over a number of decades; using plasma to replace ADAMTS 13, typically via plasma exchange over many hours and immunosuppression for immune mediated TTP and plasma infusion for congenital TTP. The risks of plasma include reactions, anaphylaxis and potential pathogen transfer, in addition, those not yet identified. This can be reduced by the use in some countries of plasma that has undergone viral (and prion) inactivation steps. With either therapeutic approach, the maximum concentration of ADAMTS 13 activity that can be achieved is limited by the volume of plasma that can be safely infused. However, while replacing ADAMTS 13, other coagulation factors, including VWF and Factor VIII, will be infused.

Completed, is the first in human, phase 1 study of recombinant ADAMTS 13 protein, developed in CHO cells, confirming its use was safe and efficacious in congenital TTP patients [4]. Fifteen patients received a single dose of BAX930 in an escalating dosing schedule. Following a 40 U/kg dose (highest dosing cohort), a maximal concentration of approximately 90 U/dL was achieved with a terminal half-life of approximately 2-3 days. This is comparable to the limited data documented with plasma therapy [5]. There were no drug associated antibodies or significant side effects related to the drug. There was also a demonstrable reduction in the UL VWF multimers and an increase in intermediate multimers, generating a physiological milieu.

What does this mean for the future of TTP treatment? Recombinant ADAMTS 13 may avoid the high adverse reaction rates with standard plasma as well as pathogen transfer risks. In congenital TTP, delivery of plasma infusion over a lifetime can be difficult because of repeated reactions. The risk is an inability to adequately treat patients because of significant side effects from therapy and the result is end organ damage affecting vital organs such as the heart, brain or kidneys. Recombinant ADAMTS 13 provides a pure replacement of the missing enzyme. The smaller infusion volume may allow for higher ADAMTS 13 activity levels to be achieved to more optimally treat the disease. Small volumes of this specific enzyme and ease of administration should significantly improve the immediate and longer-term impact of disease.

Potential future roles for recombinant ADAMTS 13: Currently a single volume plasma exchange in iTTP involves 3-5 litres of plasma, insertion of a central venous catheter and associated potential complications. Planned studies in iTTP aim to demonstrate the utility of replacement strategies with small volumes of pure ADAMTS 13 protein, with the potential to transform the treatment of this life-threatening condition. High VWF levels (and consumption of ADAMTS 13) are seen in a number of medical conditions, most notably stroke[6] and cardiovascular disease [7]. Further work will be required to establish the possible role of recombinant ADAMTS 13 in these conditions.

The development and use of recombinant ADAMTS 13 in TTP is a landmark achievement in haemostasis and thrombosis in this decade. It has the potential to offer a pure, safe therapy for congenital and immune mediated TTP that may revolutionise treatment pathways: once the trials of safety and efficacy are complete. The significant success from the Phase I data will allow progress of these trials and important improvements, ultimately, in patient care.
